# Effect of goal-directed therapy on outcome after esophageal surgery: A quality improvement study

**DOI:** 10.1371/journal.pone.0172806

**Published:** 2017-03-02

**Authors:** Denise P. Veelo, Mark I. van Berge Henegouwen, Kirsten S. Ouwehand, Bart F. Geerts, Maarten C. J. Anderegg, Susan van Dieren, Benedikt Preckel, Jan M. Binnekade, Suzanne S. Gisbertz, Markus W. Hollmann

**Affiliations:** 1 Department of Anaesthesiology, Academic Medical Center, Amsterdam, The Netherlands; 2 Department of Surgery, Academic Medical Center, Amsterdam, The Netherlands; 3 Department of Intensive Care, Academic Medical Center, Amsterdam, The Netherlands; Baylor College of Medicine, UNITED STATES

## Abstract

**Background:**

Goal-directed therapy (GDT) can reduce postoperative complications in high-risk surgery patients. It is uncertain whether GDT has the same benefits in patients undergoing esophageal surgery. Goal of this Quality Improvement study was to evaluate the effects of a stroke volume guided GDT on post-operative outcome.

**Methods and findings:**

We compared the postoperative outcome of patients undergoing esophagectomy before (99 patients) and after (100 patients) implementation of GDT. There was no difference in the proportion of patients with a complication (56% vs. 54%, *p* = 0.82), hospital stay and mortality. The incidence of prolonged ICU stay (>48 hours) was reduced (28% vs. 12, *p* = .005) in patients treated with GDT. Secondary analysis of complication rate showed a decrease in pneumonia (29 vs. 15%, *p* = .02), mediastinal abscesses (12 vs. 3%, *p* = .02), and gastric tube necrosis (5% vs. 0%, *p* = .03) in patients treated with GDT. Patients in the GDT group received significantly less fluids but received more colloids.

**Conclusions:**

The implementation of GDT during esophagectomy was not associated with reductions in overall morbidity, mortality and hospital length of stay. However, we observed a decrease in pneumonia, mediastinal abscesses, gastric tube necrosis, and ICU length of stay.

## Introduction

Esophagectomy is a high-risk surgical procedure with a morbidity-rate of up to 60% and a 30-day mortality rate ranging between 3 and 5%.[1,2] This high morbidity consists mainly of pulmonary complications, and anastomotic breakdown. Accordingly, these procedures are associated with a long hospital stay and high healthcare costs.[1,3]

Fluid management during thoracic surgical procedures has mainly focused on restricting fluid administration in order to prevent pulmonary complications.[4–7] A reduction in pulmonary complications has also been reported for esophageal surgery in small retrospective studies.[4,6] Of note, a too restrictive approach seems to increase the possibility of other post-operative complications, like anastomotic dehiscence, cardiac ischemia and kidney failure.[8]

Goal-directed therapy (GDT) aims to optimize fluid administration by using objective hemodynamic variables such as predictors of fluid responsiveness, stroke volume (SV) or cardiac output (CO). GDT has been shown to reduce post-operative complications in high-risk surgery patients.[9–11] Although a few GDT studies have included several patients undergoing esophagectomy, we are not aware of any clinical study focusing specifically on this patient population.[12,13] We initiated a quality improvement program to use GDT as a new standard of care for all patients undergoing esophagectomy at our institution. Our aim was to investigate whether a SV guided GDT improves the postoperative outcome of patients undergoing esophageal surgery.

## Methods

### Design

We performed a single-center observational before after study in a tertiary referral center (Academic Medical Center, Amsterdam, The Netherlands). The medical ethical committee (METC Academic Medical Center Amsterdam) approved the protocol and the requirement to obtain informed consent was waived (March 2013). Reporting of data was done in accordance with the STROBE guideline for observational trials.[14]

The study consisted of 3 phases. Study phase 1 represents the standard treatment group (the standard group) including all consecutive esophagectomy patients operated from January 2012 to April 2013. Study phase 2 represents a 2 month period used to introduce the GDT protocol, train anesthesiologists and adjust the GDT protocol if necessary (May and June 2013). Patients in phase 2 (N = 8) were excluded from analysis. In study phase 3 (GDT group) esophageal surgery patients were treated according to the GDT protocol (July 2013 to November 2014).

All patients undergoing an elective open or minimally invasive transhiatal or transthoracic esophagectomy with gastric tube reconstruction were elegible for inclusion. We excluded patients for which the FloTrac could not be employed (such as e.g., patients with pre-existing severe arrhythmia), patients undergoing isolated esophageal salvage surgery and came for neo-oesophagus reconstruction only (stage T4b tumor with extensive chemoradiotherapy) and patients in whom surgery was stopped prematurely because of the presence of metastases.

### Outcome parameters

All data were extracted from our Patient Data Management System (PDMS). Data from phase 1 (defined as the standard group) were extracted from an already existing surgical database. Patients’ characteristics, surgical outcome and oncologic results were prospectively collected. The intervention group from phase 3 was prospectively collected. The primary outcome parameter was total morbidity, i.e. the number of patients with a complication. Secondary endpoints were: individual post-operative complications grouped as pulmonary, surgical and other. Surgical complications were grouped according to the DINDO classification in stage 3a or lower, or 3b or higher, respectively. Follow up was a maximum of 30 days; Details and of definitions are shown in text A of S1 Appendix. [15–19] Postoperative interventions, e.g. re-operations, re-intubations, radiological or endoscopic interventions were determined. Other outcome parameters included: length of ICU and hospital stay, mortality rate, readmission to ICU, post-operative fluid balance, fluid balance at discharge to ward, use of inotropes and vasopressors intra-operatively and post-operatively.

### Procedures

#### Pre-operative preparations

All patients that were malnourished or suffered weight loss due to the tumor were treated by a dietist prior to the operation (see text B in S1 Appendix). Chemoradiotherapy was started as soon as possible and the operation was planned 6–10 weeks after completing the therapy. On the operating day, patients were fasted for solid foods for six hours and clear fluids for two hours. Two hours prior to surgery two sachets (150 ml each) of preOp (Nutridrink compact (Nutricia, the Netherlands) or lemonade (300 ml) were given.

#### Anesthesia procedure

A thoracic epidural was inserted at level Th5/6 or 6/7. Contra-indications for an epidural catheter placement were patient refusal, the use of anticoagulants and/or a platelet inhibitors as described in the current ESA guidelines.[20] General anesthesia was induced with standard dosages of intravenous Propofol, Sufentanil and Rocuronium. General anesthesia was maintained with Sevoflurane. In the absence of an epidural catheter or in case of epidural failure, multimodal analgesia was employed according to our protocol including esketamine and lidocaine.

#### Surgical procedures

Surgical procedures included open or (thoraco)laparoscopic transthoracic or transhiatal esophageal resection with a gastric tube reconstruction and a cervical or an intrathoracic anastomosis. The choice for the surgical procedure depended on patient and tumor characteristics; details of the procedures have been described previously.[1, 21] The surgical procedure was performed by two surgeons in both the standard as well as the GDT population (MvBH and SG).

#### Hemodynamic monitoring

Standard monitoring consisted of electrocardiogram (EKG), heart rate (HR), systolic (SBP), mean (MAP) and diastolic blood pressure (DBP), pulsoximeter oxygen saturation (SpO2), end tidal CO_2_ (etCO_2_), insufflation pressure and mean airway pressure. Hemodynamic parameters were measured using a Flotrac sensor and displayed on the EV1000 monitor (Edwards, Irvine (CA), USA) including: stroke volume (SV), stroke volume variation (SVV), DBP, SBP and MAP, systemic vascular resistance (SVR), cardiac output (CO), cardiac index (CI). At every stage of the operation the pressure reference was levelled at the heart. Data were extracted from our patient data management system, Metavision Suite version 3.0, *i*MD*soft*, Hannover, Germany.

#### Control (Standard group)

Patients in the standard group were not treated with a GDT protocol. Baseline hemodynamic monitoring consisted of HR, SBP, and MAP. Single hemodynamic goal was a MAP of > 65 mmHg or < 20% change from baseline MAP in case of pre-existing hypertension. Patients received fluids (colloids and or crystalloids) as deemed necessary.

#### GDT protocol and hemodynamic goals

**Fig 1:**
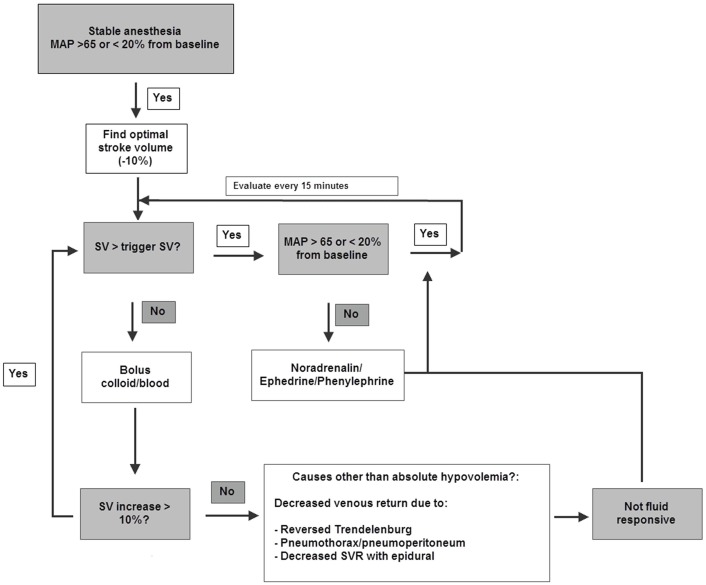
Intraoperative GDT algorithm. During the operation additional colloid boluses were given only when SV declined more than 10% below optimum (trigger SV). If SV fell below trigger during periods of decreased venous return such as pneumoperitoneum and reversed Trendelenburg and did not increase after one or two fluid boluses, (and no bleeding existed), the patient was considered not fluid responsive. Other hemodynamic goals were: MAP > 65 mmHg or < 20% change from baseline MAP. Hypotensive episodes were initially treated with Phenylefrine and/or Ephedrine. If lasting, Norepinephrine infusion was started.

An intraoperative GDT protocol was installed. Directly after induction of anesthesia, when anesthesia and hemodynamic conditions were stable, the need for giving fluids was determined using a SV-targeted GDT protocol (Fig 1 and Fig A in S1 Appendix). One or more boluses of 250 ml tetraspan 6% were administered to determine optimal SV defined as the last SV that increased by more than 10% after a fluid bolus, resembling the upper inflection point of the patients individual Starling curve. During the operation further colloid boluses were given only when SV declined more than 10% below optimum (trigger SV). Other hemodynamic goals were: MAP > 65 mmHg or less than 20% change from baseline MAP. Hypotensive episodes were treated with Phenylephrine, Ephedrine and Norepinephrine. Compliance to the protocol was recorded every 15 minutes (see text C in S1 Appendix).

#### Postoperative management

In PACU and ICU, fluid management was at the discretion of the attending recovery physician and nurses. Both groups were treated according to the same standardized clinical pathway (see text B in S1 Appendix).

### Statistical analysis

Continuous normally distributed variables are expressed as means (95%-confidence interval), and non-normally distributed continuous variables are expressed as medians (interquartile ranges). Categorical variables are expressed as numbers and percentages. An independant sample t-test was used to test differences between groups for continuous normally distributed variables, a Chi-square test was used for categorical data to test for differences between groups. When data were not normally distributed, a Mann-Whitney U test was used to analyze differences between groups. An ordered logistic regression model was conducted to examine the effect of GDT implementation on morbidity (0, 1, 2 and > 3 complications) corrected for potential confounders. Three potential confounders for which an adjustment was made in the ordered logistic regression model were: type of surgery (open versus minimally invasive surgery (scopic) and transthoracic vs. transhiatal surgery), and location of anastomosis (cervical vs. transthoracic). A multiple linear regression model was constructed for intraoperative and cumulative fluid balance as outcome by group allocation (standard vs. GDT) –as predictor corrected for potential confounders. Potential confounders for which an adjustment was made were: presence of epidural analgesia and type of surgery (open vs. minimally invasive surgery, transhiatal vs. transthoracic surgery. Odds ratios and 95% confidence intervals are reported.

Statistical significance is considered to be at *p* = 0.05. When appropriate statistical uncertainty will be expressed by the 95% confidence levels. Data were analyzed using IBM SPSS Statistics for Windows, Version 22.0 (Armonk, NY: IBM Corp.) and with R the statistical package (R Core team 2016).

## Results

### Demographics

**Fig 2:**
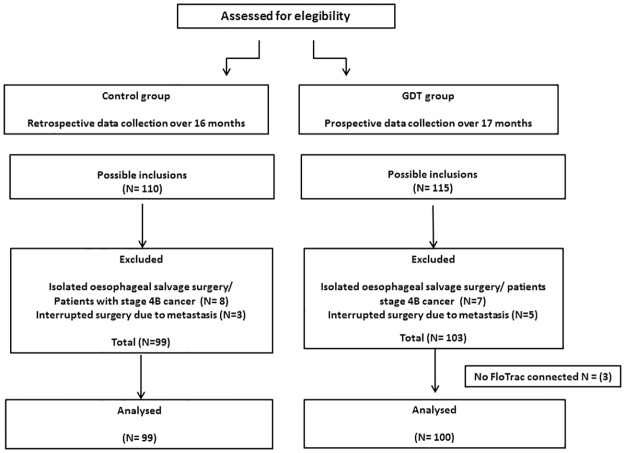
Consort diagram. We excluded patients where FloTrac was not connected (because of arrhythmia), those came for isolated esophageal salvage surgery with neo-esophagus reconstruction only (stage T4b tumor with extensive chemo-radiotherapy) and those in whom surgery was stopped prematurely because of the presence of metastases.

We included in the standard group 99 and in the GDT group 100 esophagectomy patients for analysis (consort diagram, Fig 2). Table 1 describes demographics and surgical characteristics of both populations. Two-third of the included population received a complete minimally invasive procedure. More patients in the GDT group received a minimally invasive procedure for both the transhiatal procedure as the transthoracic with intra-thoracic anastomosis procedure (*p* < .01). More patients received epidural analgesia in the standard group (*p*< .01).

**Table 1:** Patient demographics.

Demographics	Standard (N = 99)	GDT (N = 100)	*p*-value
Male	72 (73)	77 (77)	.52
Age	64 (62–66)	64 (62–65)	.85
Cardiovascular Disease	48 (49)	38 (38)	.15
COPD	7 (7)	6 (6)	.78
Diabetes Mellitus type 1 or 2	13 (13)	11 (11)	.67
BMI	26 (25–27)	26 (25–27)	.78
Creatinine (mmol/l) pre-operative	76 (72–80)	77 (73–80)	.80
**ASA classification**			
I	22 (22)	17 (17)	.57
II	68 (69)	71(71)	
III	9 (9)	12 (12)	
**Tumor characteristics and treatment**			
- pTNM stage*			
pT1-2	36 (36)	44 (44)	.81
pT3-4	37 (37)	34 (34)	
pyTx	22 (22)	19 (19)	
pN0	62 (63)	63 (63)	> .99
pN+	34 (34)	34 (34)	
M0	95 (96)	96 (96)	.84
Other**	3 (3)	3 (3)	1.0
Neo-adjuvant chemo radiation	82 (82)	85 (85)	.68
**Operation; type of resection**			
**- Transhiatal**			
Open	13 (12)	2 (2)	.003
Laparoscopic	9 (8)	16 (16)	
**- Transthoracic**			
Open	9 (9)	10 (10)	.92
Thoracolaparoscopic	68 (64)	72 (72)	
*- Cervical anastomosis*	60 (60.6)	38 (38)	<.001
*- Intrathoracic anastomosis*	8 (8)	34 (34)	
Epidural	94 (95)	82 (82)	.007

Data on patient demographics and type of surgery. For the minimally invasive transthoracic approach the number of patients that received a cervical vs. an intra-thoracic anastomosis are shown. GDT; goal-directed therapy, BMI; body mass index, COPD; chronic obstructive pulmonary disease.

* TNM stage post-surgery pathology. Incidence of T0-1, N0-1 and Mo are compared to that of T2-4, N2-3 and M1; Data are in numbers (%) or mean (95CI).

** Other: defined as benign, recurrence, salvage or high grade dysplasia.

### Morbidity outcome

Total morbidity was not different between groups. Fifty-six patients in the standard group had one complication as compared to 54 in the GDT group (*p* = .82). Table 2 shows information on the frequency and sum of complications by 0,1,2 and ≥ 3 incident categories. Ordered logistic regression showed that although type of surgery (open versus minimally invasive surgery, and transhiatal vs. transthoracic surgery) were significant predictors they did not change the primary determinant of this analysis, i.e. the use of goal directed therapy, *p* = .89. So GDT was not a predictor of morbidity in terms of 0, 1, 2 or >3 complications, (Table 3 and fig A in S2 appendix). There was no difference between groups in DINDO classification > 3b, i.e 28% vs. 21%, *p* = .31, respectively. Subanalysis on type of complications showed a significant lower incidence in pneumonia, mediastinal abscesses and gastric tube necrosis in patients treated with GDT (Table 4).

**Table 2:** Overall morbidity; frequency and sum of complications by incident categories.

	Standard		GDT		Total	
Incident categories	Freq	Sum*	Freq	Sum*	Freq	Sum*
0 complications	43	0	46	0	89	0
1 complication	21	21	23	23	44	44
2 complications	11	22	17	34	28	56
3 or more complications	24	111	14	51	38	162
**Sum**	99	155	100	107	199	262

* Actual number of incidents that occurred within the incident category.

**Table 3:** Ordered logistic regression analysis.

		95% CI		
	OR	Lower	Upper	*p* value
**Model 1)** Included all the potential predictors beside the primary determinant (GDT) for complications (morbidity)				
**GDT_yes_no**	**0.96**	**0.55**	**1.68**	**0.89**
Open_Minimally invasive	0.35	0.16	0.74	0.006
Thocr_Ttocr	0.34	0.16	0.70	0.004
ProxAnas.c	0.78	0.39	1.52	0.46
**Model 2)** The same as model 1 but without the location of anastomosis variable (ProxAnas.c)				
**GDT_yes_no**	**0.87**	**0.51**	**1.46**	**0.59**
Open_Minimally invasive	0.37	0.18	0.76	0.007
Thocr_Ttocr	0.42	0.20	0.83	0.014
**Model 3)** Model which includes GDT as a single predictor				
**GDT_yes_no**	**0.80**	**0.48**	**1.33**	**0.39**

Interpretation of the model: for one unit increase in goal directed therapy (GDT), i.e. going from No GDT to GDT is yes, the Odds of zero complications versus one, two or at least three complications combined are 0.96 (95% CI 0.55 to 1.68), *p* = 0.89, given that all other variables in the model are held constant. Although type of surgery (open versus minimally invasive surgery), and type of surgery (transhiatal surgery (thocr) vs. transthoracic surgery (ttocr)) showed to be significant predictors they did not change the primary determinant of this analysis, i.e. the use of goal directed therapy. So GDT is not a predictor of morbidity in terms of 1, 2 or at least 3 complications.

**Table 4:** Secondary analysis of post-operative complication rates.

Pulmonary complication	Standard (N = 99)	GDT (N = 100)	% Difference (95% CI), *p* value
Pulmonary complications*	36 (36)	29 (29)	7 (-7 to 21), .27
*Pneumonia*	29 (29)	15 (15)	14 (3 to 26), .02
*Pneumothorax*	11 (11)	8 (8)	3 (-6 to 12), .55
*Atelectasis*	1 (1)	1 (1)	0 (-3 to 3), .99
*Pleural fluid*	14 (14)	15 (15)	-1 (-11 to 10), .86
**Surgical complications**			
Surgical complications **	34(34)	33 (33)	1 (-13 to 15), .84
*Post-Surgical Bleeding*	0	2	-2 (-6 to 2), .48
*Mediastinal abscess*	12 (12)	3 (3)	9 (1 to 18), .02
*Abdominal abscess*	1 (1)	1 (1)	0 (-3 to3), .99
*Anastomotic leakage*	17 (17)	15 (15)	2 (-9 to 13), .68
*Chylus leakage (abdominal + thoracic)*	14 (14)	6 (6)	8 (-1 to 17), .06
*Gastric tube necrosis*	5 (5)	0	5 (0 to 10), .03
*Wound infection*	1 (1)	3 (3)	-2 (-7 to 3), .32
*Sepsis*	10 (10)	5 (5)	5 (-3 to 13), .17
*Leaking or dislocated jejunum fistula*	7 (7)	3 (3)	4 (-3 to 11), .19
**Other**			
*Trombo-embolic event*	5 (5)	5 (5)	0 (-6 to 6), .99
*Neurological events*	3 (3)	2 (2)	1 (-4 to 6), .64
*Cardiac events*	25 (25)	23 (23)	2 (-11 to 15), .71
*Elevation of creatinine of > 50%*.	16 (16)	16 (16)	0 (-10 to 11), .98

Data are in numbers (%) and percentage difference (95CI). GDT; goal-directed therapy.

* The presence of one or more pulmonary complication.

** The presence of one or more surgical complication.

### Post-operative (re-)interventions

Fewer patients needed to be re-intubated in the GDT group: 19% in the standard versus 10% in the GDT group, although statistical significance was not reached (*p* = 0.07). There were no differences (standard vs. GDT) in re-operations 19 vs. 13%, (*p* = .33), proximal gastric tube disconnections made, 11 vs. 5%, *p* = .19 and radiological and/or endoscopic interventions 25 vs. 26% (*p* = 1.0).

### Length of stay and mortality

Median post-operative ICU or PACU (post anesthesia care unit) stay (first admittance) was higher in the standard group (median 22 (IQR 19–64) vs. 19 (IQR 17–40) hours (*p* = .001). The number of patients with a PACU or ICU stay of more than 48 hours was also higher in the standard group, (28 vs.12%), *p* = .005. There was no difference (standard vs. GDT) in incidence of ICU re-admission (23 vs. 23%, *p* = 1.00) or median hospital length of stay 12 (IQR 9–24) days vs. 13 (IQR 10–25), *p* = .55. The in-hospital mortality rate was not significantly different 6 vs 3%, *p* = .33.

### Fluid balance and use of vasopressors

**Fig 3:**
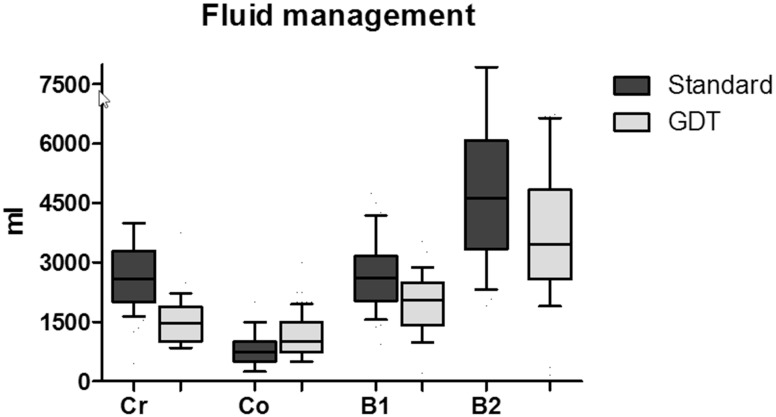
Box and whisker plot of fluid balances and type of fluids used in the GDT and the standard group. The use of crystalloids (Cr), colloids (Co), intra-operative (B1) fluid balance and cumulative balance at dismissal to the ward (B2) are shown. GDT; goal-directed fluid therapy. Boxes represent interquartiles with whiskers showing 10–90% ranges.

Median intra-operative blood loss (standard vs. GDT) was 200 (IQR 50–400), vs. 200 (IQR 200–400) ml, (*p* = .11)). Median fluid balance at the end of the operation was 2625 (IQR 2035–3167) vs. 2052 (IQR 1457–2497) ml (*p*<0.001). Post-operative fluid infusion was not different between groups, namely, 1624 ml vs 1560 (*p* = .27), cumulative fluid balance at dismissal to the ward was 4633 (IQR 3327–6091) vs. 3490 (IQR 2575–4860) ml for standard vs. GDT, respectively (*p*<.001). More colloid 750 (500–1000) vs. 1000 (750–1500) ml and less crystalloid fluids (2500 (2000–3250) vs. 1250 (1000–1750) ml) were used during anesthesia when applying GDT, (*p*<.001, Fig 3). Colloid dosages adjusted for weight were 9 (6–14) and 14 (9–20) ml/kg, in the standard vs. GDT group respectively. In a multivariable linear model, GDT was an independent predictor for fluid balance at the end of the operation, *p* <.001. GDT was also a predictor for the cumulative amount of fluids received at the time of discharge to the ward (*p*<.001, see S2 Appendix).

Intraoperative cumulative norepinephrine dose was higher in the GDT group 0.9 (IQR 0.4–1.6) vs. 1.6 (IQR 0.9–2.5) mg but more phenylephrine was used in the standard group, 300 (IQR 0–600) vs. 100 (IQR 0–300) mcg (*p*<.001). There was no difference in the use of ephedrine between groups, 10.0 (0–20.0) vs. 12.5 (5.0–25.0) mg, (*p* = .39).

### Compliance to protocol

Anesthesiologist’s adherence to the GDT protocol was high. Median compliance rate was 96 (90–100)%. Compliance > 90% was seen in 77 patients (77%). Sixteen patients (16%) reached compliance between 70 and 90%. In only 6 (6%) patients compliance was below 50% and the protocol was or could not be followed. In one case compliance could not be assessed because data were lost. Mean percentage of time that actual SV was above target SV was 79 (SD 20) %.

## Discussion

This is the first study assessing the effects of GDT implementation on the postoperative outcome of patients undergoing esophageal surgery. The implementation of GDT was not associated with a decrease in overall morbidity but we observed a decrease in the rate of pneumonia, mediastinal abscesses and gastric tube necrosis in secondary outcome analysis, as well as a decrease in patients needing a ICU stay of >48 hours.

Recent meta-analyses on the effect of GDT in the perioperative setting have shown reductions in morbidity.[10,11,22] especially in surgical site infections and respiratory failure.[9,23,24] In contrast to other studies, we observed no decrease in overall morbidity, hospital stay or mortality. The decreased incidence in pneumonia and mediastinal abscesses is in line with those earlier reports.[9–11,22–24] The incidence of a ICU stay > 48 hrs was decreased in patients treated with GDT. As logistics and postoperative care was not changed this is most likely due to a lower incidence of mediastinal abscess, necrosis and pneumonia in the GDT group, which may impact ICU stay in those patients. Although Median ICU stay was also decreased but only by 2 hours, which is consistent with other studies.[25,26] We failed to show a reduction in anastomotic leakage, which is an important contributor to morbidity in this population. The latter is in line with the data reported by Wei et al. who did not find any relationship between fluid balance and anastomotic leakage.[27] On this aspect, this patient population may not be comparable to other groups of patients undergoing abdominal surgery. During esophageal resection, multiple arteries are ligated and the blood supply of the newly formed gastric tube depends solely on the right gastro-epiploic artery. This leaves the fundus of the stomach where the future anastomosis is formed dependent on passive diffusion of blood through the gastric wall. Therefore, it is likely that anastomotic leakage cannot simply be prevented by restricting or optimizing fluid administration, as surgical and anatomical factors may play a more important role.

Two-third of our patients underwent minimally invasive surgery. There are no data describing the use of GDT in thoraco-laparoscopic surgery. Some experience with GDT during laparoscopy has been made in patients for colectomy.[28,29] However, these procedures are clinically not comparable to esophagectomy. For our GDT algorithm, we chose a SV-guided algorithm adjusting the Kuper protocol. The Kuper protocol is part of official guidelines in several countries and has been successfully used in a large quality improvement project.[30] Although the use of SVV has been investigated in esophagectomy patients,[12,13] this target would be unreliable during open chest surgery and pneumoperitoneum.[31,32] By using a SV based protocol we were able to use the same protocol in both open and minimally invasive patients. As inflation pressures may have an effect on SV, we did not give more fluids if SV did not increase and examined whether vasopressors or inotropes would be a better choice to increase venous return as shown in Fig 1. More research is needed to determine the benefits of GDT during thoraco-laparoscopic surgery.

Intra-operative fluid balance was lower in the GDT group. Restricting fluids reduced pulmonary complications in thoracic surgery, especially when using one lung ventilation.[4–6] An observational study of Wei et al. showed that cumulative fluid balance until 48 hours post-esophagectomy was correlated with cardiac and pulmonary complications.[27] Our study also showed a decrease in the post-operative fluid balance of the GDT group. This might have additionally influenced the difference in pulmonary complication rate between groups.

Depending on the study, implementation of GDT has been reported to either reduce or increase fluid balance.[22,33–35] The observed effect of GDT varies with the population studied, the type of surgery, pre-GDT fluid habits (liberal or restrictive), type of fluid and the hemodynamic algorithm used. Therefore, the strength of GDT might not only rely on optimizing the amount of fluids administered but also in the timing of fluid administration.

We used colloids to optimize preload and this may have influenced fluid balance in the GDT group as well. Several studies describe a decrease in fluid balance and a higher increase of SV or CO when using a colloid based GDT protocol as compared to a crystalloid based protocol.[36–38] This might especially be advantageous in thoracic surgery. Although colloids may be contraindicated in patients with sepsis, kidney failure or burns [39], there is no literature that convincingly shows an increased risk when using colloids in a non-septic (surgical) population.[40] In addition, we did not find a higher creatinine in our GDT patients. Likewise, no differences in complication rate or other outcome variables have been found with the use of colloids in earlier studies.[37,38] The use of noradrenaline increased while the use of phenylephrine decreased with the GDT protocol. We did not specifically change practice in vasopressor use during the study. Phenylephrine may reduce CO and SV in normovolemic patients and it is likely that this fact has led to reduced use. The protocol also takes into account other variables such as SVR trending. Accordingly, anesthesiologists may have decided that increasing noradrenaline instead of giving a bolus phenylephrine was a more rational approach.

There are certain limitations of this study. First, this study was set up as a before- after study. Accordingly, the benefits observed may not be exclusively related to the implementation of GDT. Disadvantages of before- after studies are the risk of bias in patient selection, the influence of time on surgical techniques used and surgical experience. In our study there was an imbalance in the surgical techniques used and the presence of epidural analgesia, possibly influencing outcome and the amount of fluids used. We adjusted for this confounding influence in a multivariate analysis and found that correcting for surgical factors did not change outcome and that no correlation between epidural use vs. fluid balance existed. The before-after design also has advantages. It may better represent daily clinical practice with its lack of controlled circumstances, varied patient population and implementation barriers. In addition, the standard arm is not influenced by the interventional arm by an effect of training or the Hawthorne effect: because you train clinicians for GDT you will inevitably modify their believes and practice in the control group (training effect); because they are observed, they inevitably behave differently and the so-called control group is not a real standard of care group. Lastly, if multiple RCTs and meta-analyses have shown consistent positive effects in strict clinical research setting, the before and after study design may be a good alternative to evaluate the impact of an intervention outside a research setting. Due to the fact that the control group was an already existing prospectively collected dataset, the patient characteristics and data that were available were not specifically designed for this study which may have influenced the results. However, all outcome variables for this study were available in the database.

Another limitation is that GDT was limited to intra-operative use only. We might have found a more profound reduction in complication rate when GDT had been used also in the postoperative phase. We did not report fluid infusion on the surgical ward. Both groups were treated with ERAS and differences in treatment were not expected. A recent meta-analysis has shown that avoidance of fluid overload and managing patients under enhanced recovery protocols reduces the magnitude of benefit of GDT. Accordingly, future studies should also take into account fluid infusion on the ward.[41]

## Conclusions

The implementation of GDT during esophagectomy did not reduce overall morbidity, mortality and hospital length of stay. However, pneumonia, mediastinal abscesses, the proportion of patients staying > 48h in the ICU and fluid balance were lower in the GDT group. To our opinion this makes larger (randomized) studies necessary to reveal possible benefits with a higher reliability. The economic impact of GDT remains to be determined.

## Supporting information

S1 Appendix Detailed information on methods. Fig A. Flowchart showing the steps of finding optimal stroke volume. The optimal filling status of the patient was reached by giving one or more boluses of 250 ml tetraspan 6% (step 1) until SV could be no longer increased (step 2). Optimal SV was defined as the last SV that increased by more than 10% after a fluid bolus. During the operation further colloid boluses were given only when SV declined more than 10% below optimum (trigger SV, step 3).
(DOC)

S2 Appendix Detailed information on regression analyses. Fig A. Distribution of potential predictive values by number of complications. GDT; goal directed therapy 1 = yes,2 = no, thocr: transhiatal esophagus resection = 2; Ttocr: transthoracic esophagus resection = 1. Location of proximal anastomosis, 2 = cervical, 3 = transthoracic.
(DOCX)
