# Supplementary material for: Effect of goal-directed therapy on outcome after esophageal surgery: A quality improvement study
Source: PLoS One. 2017 Mar 2;12(3):e0172806. doi: 10.1371/journal.pone.0172806 (PMC5333843; doi:10.1371/journal.pone.0172806)

**S1 Appendix**

1. **Definitions** [15-19]

Total morbidity; the presence of a complication.

Chronic obstructive pulmonary disease (COPD); diagnosed according to the GOLD classification. Cardiovascular disease; including hypertensive disease with medication, ischemic cardiac disease and peripheral vascular disease. Diabetes mellitus; Type 1 or 2, with need for medication. The composite endpoint “one or more complications’; the % of patients that had zero complications as compared to those with one or more pulmonary or surgical complication.

Pulmonary complications: Atelectasis; clinically relevant atelectasis (lobar collapse on chest radiograph). Pneumonia; radiological confirmation of an infiltrate, combined with positive cultures and elevated temperature (above 38.5 degrees Celsius or elevated leucocytes or elevated CRP. Antibiotics were started pre-emptive and was later guided by bacterial gram stain and culture. 3) Pneumothorax; collection of air between the visceral and parietal pleural surfaces, requiring drainage. Pleural effusion; collection of fluid between the visceral and parietal pleural surfaces, requiring drainage, and acute respiratory failure (partial pressure of arterial oxygen <60 mm Hg while breathing ambient air).

Surgical complications: Post-operative surgical bleeding was defined as post-operative blood loss requiring blood transfusion and/or leading to hemodynamic instability. Mediastinal abscess was scored when an abscess was identified on radiologic imaging or intraoperative visualization requiring interventional or antibiotic treatment. Anastomotic leakages were recorded when they were clinically manifest and confirmed by physical examination, radiologic imaging or intraoperative/endoscopic visualization. Gastric tube necrosis was scored in case of intraoperative/endoscopic confirmation of ischemic changes in the gastric conduit. Chylothorax was recorded when elevated levels of triglycerides in intra-thoracic fluid (>1 mmol per liter [89 mg per dL]) were found). Wound infection was defined as a contaminated wound requiring any type of intervention. Sepsis was scored in case hemodynamic instability of inflammatory/infectious origin.

Clavien-Dindo classification: Grade 1: Any deviation from the normal postoperative course without the need for pharmacological treatment or surgical, endoscopic and radiological interventions. Grade 2: Requiring pharmacological treatment with drugs other than such allowed for grade I complications. Blood transfusions and total parenteral nutrition are also included. Grade 3: Requiring surgical, endoscopic or radiological intervention; 3A: intervention not under general anesthesia. 3B: intervention under general anesthesia. Grade 4: Life-threatening complication (including CNS complications)‡ requiring IC/ICU-management. 4A: single organ dysfunction (including dialysis). 4B: multi organ dysfunction. Grade 5: death.

Other complications: Thrombo-embolic events were recorded when a pulmonary embolus (embolus detected on spiral CT or a ventilation–perfusion mismatch on a lung scintigram) or deep vein thrombosis (detected on duplex ultrasound) was diagnosed. Neurological events included delirium, (poly)neuropathy and cerebrovascular events. Cardiac complications were arrhythmia (any change in rhythm on the electrocardiogram, requiring treatment), myocardial infarction (two or three of the following: previous myocardial infarction, electrocardiographic changes suggesting myocardial infarction, or enzyme changes suggesting myocardial infarction), and left ventricular failure (marked pulmonary oedema on a chest radiograph). Kidney function disorder; elevation of the pre-operative creatinine of > 50%.

1. **Preoperative and postoperative pathway**

**Preoperative preperations**

All patients that were malnourished or suffered weight loss due to the tumor were treated by a dietist prior to the operation. In our hospital the SNAQ score is used to detect preop malnutrition.[ Kruizenga HM, Seidell JC, de Vet HC, Wierdsma NJ, van Bokhorst-de van der Schueren MA. Development and validation of a hospital screening tool for malnutrition: the short nutritional assessment questionnaire (SNAQ). Clin Nutr 2005;24: 75-82.] Patients with severe dysphagia received enteral feeding by nasogastric tube. Two hours prior to surgery two sachets (150 ml each) of preOp (Nutridrink compact (Nutricia, the Netherlands) or lemonade (300 ml) were given.

**Enhanced recovery after surgery protocol (ERAS)**

Aims for the first postoperative day were: 1) nasogastric tube and chest tubes removed if not clinically contraindicated; 2) enteral feeding was started via the jejunum fistula and increased over the next days according to predefined regime. 3) start mobilisation. 4) a protocol for optimization of pain management was in place for both patients with an epidural analgesia as those without. Epidural was routinely removed after 3-4 days postoperatively. At the ward fluid administration target was 2 L per 24 hours.

1. **Goal directed fluid therapy protocol**

Intraoperatively, one or more boluses of 250 ml tetraspan 6% were given to determine optimal SV. Optimal SV was defined as the last SV that increased by more than 10% after a fluid bolus, resembling the upper inflection point of the patients individual Starling curve (Figure S1). During the operation further colloid boluses were given only when SV declined more than 10% below optimum (trigger SV). Baseline fluid maintenance (to correct for insensible loss) infusion was limited to 1 ml/kg/hr crystalloids (Sterofundin, Braun, the Netherlands). If SV decreased below the trigger during periods of decreased venous return such as pneumoperitoneum of reversed Trendelenburg and did not increase after a maximum of two fluid boluses, and in the absence of active bleeding, the patient was considered fluid unresponsive. Other hemodynamic goals were: MAP > 65 mmHg or less than 20% change from baseline MAP. Hypotensive episodes were initially treated with Phenylephrine (if SVR < 1200 dynes - sec/cm–5) and/or Ephedrine (if HR < 60 bpm) boluses. If hypotension lasted, Norepinephrine infusion was started to modulate vasotonus. Postoperatively, fluid and hemodynamic management was according to the attending anesthesiologist.

**Compliance to GDFT protocol:**

SV was recorded on paper forms. Trigger SV (Fig A) was determined and SV was collected each 15 minutes. Furthermore the infusion of a fluid bolus, amounts of inotropes needed and (changes in) vasopressors were noted. Finally, if a patient was fluid unresponsive this was also registrated. For each patient compliance to protocol was determined by evaluating the proportion of 15-minute registrations without protocol violations. A protocol violation was defined as: 1) a SV below trigger SV in combination with no bolus administered and no registration of earlier fluid unresponsiveness. 2) no SV registrated. All patients were included in the analysis independent of compliance to the protocol.

**Fig A.**


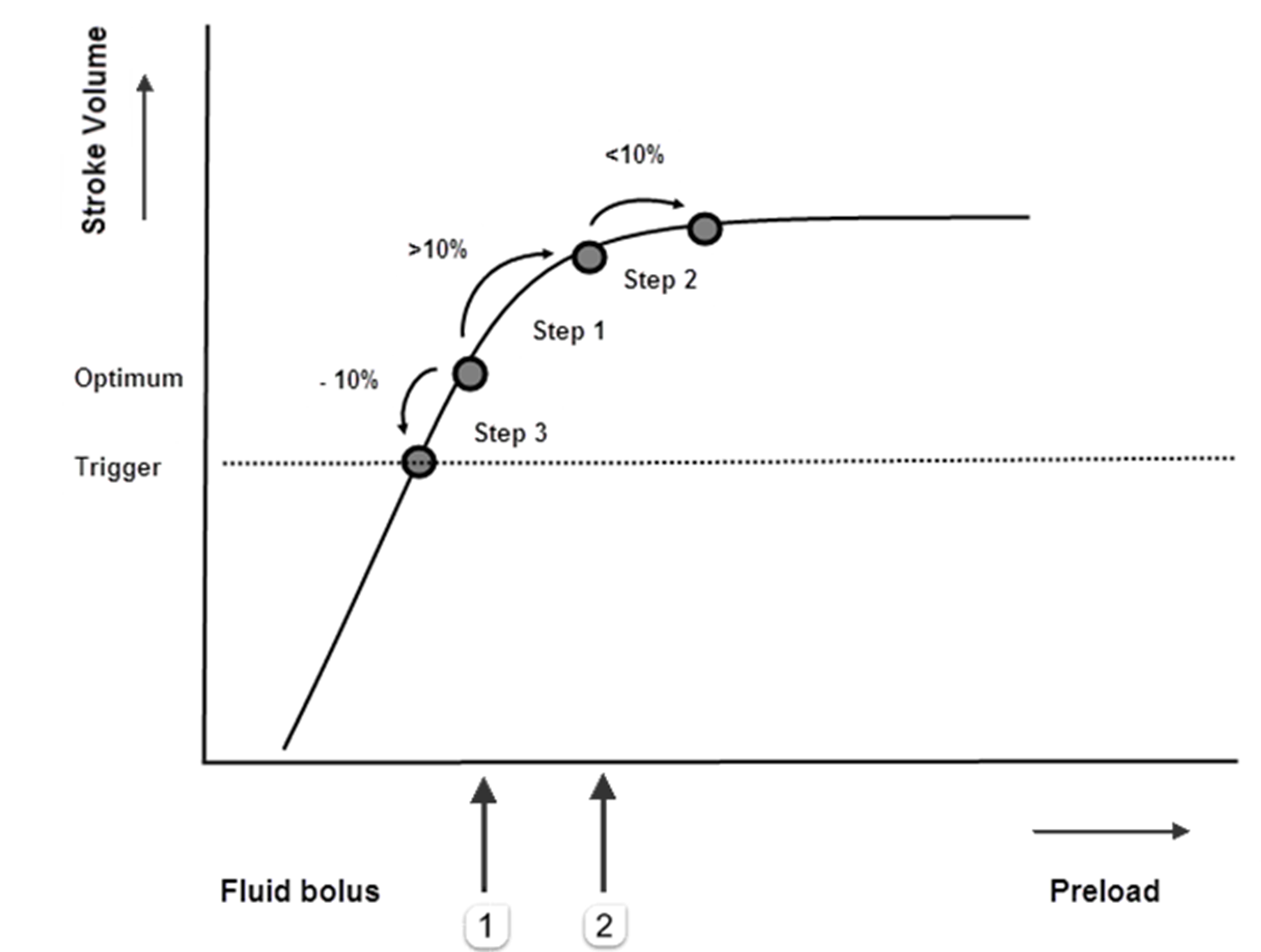

Supplement: S1 Appendix — The optimal filling status of the patient was reached by giving one or more boluses of 250 ml tetraspan 6% (step 1) until SV could be no longer increased (step 2). Optimal SV was defined as the last SV that increased by more than 10% after a fluid bolus. During the operation further colloid boluses were given only when SV declined more than 10% below optimum (trigger SV, step 3). (DOC) [file pone.0172806.s001.doc]
