# Supplementary material for: Effect of goal-directed therapy on outcome after esophageal surgery: A quality improvement study
Source: PLoS One. 2017 Mar 2;12(3):e0172806. doi: 10.1371/journal.pone.0172806 (PMC5333843; doi:10.1371/journal.pone.0172806)
Supplement: S2 Appendix — GDT; goal directed therapy 1 = yes,2 = no, thocr: transhiatal esophagus resection = 2; Ttocr: transthoracic esophagus resection = 1. Location of proximal anastomosis, 2 = cervical, 3 = transthoracic. (DOCX) [file pone.0172806.s002.docx]

**S2 Appendix**

**A. Fig A.**


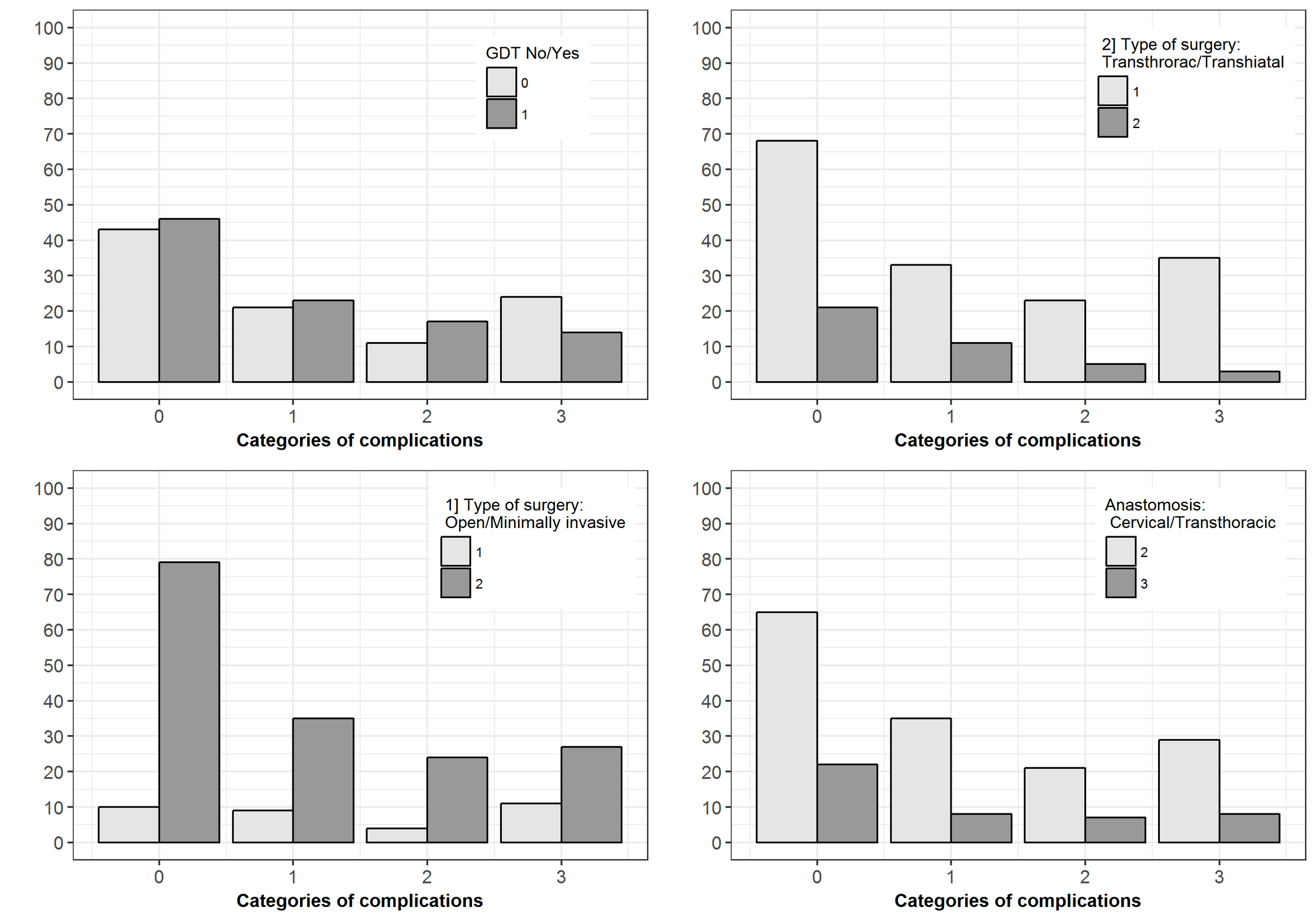


**B. Linear regression analysis**

| **Model 1)** Included all the potential predictors beside the primary determinant (GDT) for Intraoperative fluid balance. Predictors with a *p*-value >.10 were excluded from model 2. | | | | |
| --- | --- | --- | --- | --- |
|  |  | **95% CI** | |  |
|  | **Bèta** | **Lower** | **Upper** | ***p* value** |
| **GDT_yes_no** | **-701,544** | **-962,358** | **-440,729** | **0.00** |
| Open_minimally invasive | 31,755 | -321,461 | 384,972 | 0.859 |
| Thocr_Ttocr | -313,853 | -642,514 | 14,807 | 0.061 |
| Epidural yes_no | -161,852 | -567,215 | 243,512 | 0.432 |

| **Model 2)** The same as model 1 but without open_minimally invasive and epidural variables | | | | |
| --- | --- | --- | --- | --- |
|  |  | **95% CI** | |  |
|  | **Bèta** | **Lower** | **Upper** | ***p* value** |
| **GDT_yes_no** | **-719,516** | **-972,425** | **-466,607** | **.000** |
| thocr_ttocr | -319,992 | -635,532 | -4,453 | .047 |

| **Model 1)** Included all the potential predictors beside the primary determinant (GDT) for cumulative postoperative fluid balance. Predictors with a p-value >.1 were excluded. | | | | |
| --- | --- | --- | --- | --- |
|  |  | **95% CI** | |  |
|  | **Bèta** | **Lower** | **Upper** | ***p* value** |
| **GDT_yes_no** | **-894,201** | **-1739,169** | **-49,232** | **.038** |
| Open_scopic surgery | -1644,818 | -2789,144 | -500,492 | .005 |
| thocr_ttocr | -620,149 | -1684,921 | 444,623 | .252 |
| Epidural yes_no | -333,166 | -1646,433 | 980,102 | .617 |

| **Model 2)** The same as model 1 but without thocr_ttocr and epidural variable | | | | |
| --- | --- | --- | --- | --- |
|  | | **95% CI** | |  |
|  | **Bèta** | **Lower** | **Upper** | ***p* value** |
| **GDT_yes_no** | **-927,811** | **-1755,656** | **-99,966** | **.028** |
| Open_scopic | -1481,926 | -2581,654 | -382,199 | .009 |

A multivariate linear regression model was constructed for intraoperative and cumulative fluid balance by group allocation (standard vs. goal directed therapy (GDT ))–corrected for potential confounders. Potential confounders for which an adjustment was made were: presence of epidural analgesia and type of surgery (open vs. minimally invasive surgery, transhiatal vs. transthoracic surgery). GDT was an independent predictor for fluid balance at the end of the operation. Patients treated with GDT had received a mean of 720 ml less fluids than the standard group (p< .001). GDT was also a predictor for the cumulative amount of fluids received at the time of discharge to the ward

**References**

1. Software and datasets to support 'Modern Applied Statistics with S', fourth edition, by W. N. Venables and B. D. Ripley. Springer, 2002, ISBN 0-387-95457-0.

2. R Core Team (2016). R: A language and environment for statistical computing. R Foundation for

Statistical Computing, Vienna, Austria. URL https://www.R-project.org/.
